# Impact of Adjuvant Radiotherapy on Free Flap Volume in Autologous Breast Reconstruction: A Scoping Review

**DOI:** 10.3390/jcm13010217

**Published:** 2023-12-29

**Authors:** Pablo Pfister, Seraina L. C. Müller, Anna-Lena Eberhardt, Medea Rodriguez, Nadia Menzi, Martin Haug, Dirk J. Schaefer, Elisabeth A. Kappos, Tarek Ismail

**Affiliations:** 1 Department of Plastic, Reconstructive, Aesthetic and Hand Surgery, University Hospital Basel, 4031 Basel, Switzerlandmedeaelisa.rodriguez@usb.ch (M.R.); nadia.menzi@usb.ch (N.M.); martin.haug@usb.ch (M.H.); dirk.schaefer@usb.ch (D.J.S.); elisabeth.kappos@usb.ch (E.A.K.); tarek.ismail@usb.ch (T.I.); 2 Department of Radiation Oncology, University Hospital Basel, 4031 Basel, Switzerland; 3 Faculty of Medicine, University of Basel, 4031 Basel, Switzerland

**Keywords:** breast reconstruction, free flap, flap volume, adjuvant radiotherapy

## Abstract

In autologous breast reconstruction, a sufficient flap volume is fundamental to restore breast shape and ensure an aesthetic outcome. After mastectomy, postoperative irradiation is regularly indicated in the oncological treatment algorithm. When administering radiation therapy after autologous reconstruction, the tissue transferred is inherently irradiated. Although there is evidence that points to a reduction in flap volume after adjuvant radiotherapy, the data have been contradicting and inconclusive. To address this anecdotal evidence, we performed a scoping review of the current literature that addresses the effect of radiotherapy on breast flap volume. Six two-armed studies, comprising a total of 462 patients, reported on the effect of adjuvant radiotherapy on free flap volume changes. Of those, two studies found a significant negative impact of radiotherapy on free flap volume, while the other four studies did not. Reported flap volume changes ranged from no change to a reduction of 26.2%, measured up to two years postoperatively. The selected studies contain varying patient numbers, follow-up timepoints, types of flaps, and measuring methods, contributing to a relatively high heterogeneity. While we present some evidence suggesting a significant impact of adjuvant radiotherapy on breast flap volume, future studies are needed to further investigate this potential correlation.

## 1. Introduction

Current algorithms in breast cancer treatment include a personalized combination of drug therapy, surgical resection, and radiotherapy, depending on the age of the patient, cancer classification, and tumor characteristics [1]. With recent advancements in these therapeutic regimens, the population of long-term breast cancer survivors has substantially risen [2]. Consequently, there is now an increased research focus on the period following treatment. Pivotal to this phase is the patients’ quality of life, making it imperative to provide them with a robust approach that yields lasting results, not only regarding oncological outcomes [3]. 

Even though breast-conserving therapy is a valid choice for many affected women, mastectomy still remains important when it comes to treating locally advanced disease or prophylactic surgeries [4,5]. Mastectomy is indicated in 30–40% of all breast cancer patients, a majority of whom choose a reconstruction of the amputated breast [6,7,8]. When choosing the reconstructive technique, there are several options available, including alloplastic methods using tissue expanders and implants, autologous reconstruction with free tissue transfer, and a combination of both [9]. While both approaches hold valid indications, recent studies have shown that autologous reconstructions lead to higher levels of breast satisfaction and psychosocial and sexual well-being [10,11,12,13]. For free flap autologous breast reconstruction, the deep inferior epigastric perforator (DIEP) flap is the current workhorse flap [14,15]. Other harvesting options include the profunda artery perforator flap (PAP), the transverse myocutaneous gracilis flap (TMG), and the superior gluteal artery perforator flap (SGAP) [16,17,18]. Although autologous reconstruction results in increased breast satisfaction, it is important to mention that the optimal personalized breast reconstruction approach is always based on joint and informed decision making between the patient and the surgeon [19]. Important in this decision making process is the potential influence of adjuvant treatment modalities on postoperative outcomes. This gains significant importance in the setting of adjuvant radiotherapy since irradiation of the reconstructed breast has been shown to influence complication rates and aesthetic outcomes [20]. Postmastectomy radiotherapy is indicated for high-risk patients, especially for those with T3-4 disease or nodal involvement, to lower the risk of locoregional recurrence [21]. It increases local tumor control, reduces mortality, and has a relatively high benefit–risk ratio [22,23,24]. Potential downsides of radiotherapy are its late toxicities including fibrosis or the shrinking of the reconstructed tissue [25,26]. This effect could significantly impact breast symmetry and thus lead to a decrease in patient satisfaction [27]. To bypass this problem, breast reconstruction can be delayed until after radiotherapy. Evidence has shown a decrease in the number of surgical complications including volume loss, fat necrosis, and revision surgeries for delayed autologous reconstruction [28,29,30,31]. However, a delay of the reconstruction includes risks related to the alloplastic procedures, as well as the need for a multi-step procedure instead of immediate reconstruction [32]. There is still an ongoing debate about the ideal timing of reconstructive procedures, since other studies have shown that immediate autologous reconstruction is well tolerated regarding complications rates, even in the setting of planned adjuvant radiotherapy [31,33]. One argument against an immediate reconstruction is the suspected reduction in flap volume following irradiation. Despite some data indicating a decrease in flap volume following adjuvant radiotherapy, other authors have shown no significant influence [34,35,36,37,38,39,40]. Since breast symmetry, and thus flap volume, is important for the aesthetic outcome and patients’ breast satisfaction, we decided to summarize the current body of evidence through a systematic scoping literature search [41].

## 2. Methods

### 2.1. Literature Strategy

Before conducting a full literature search, we performed a pilot search in order to estimate the available literature on this topic. Due to the expected scarcity of evidence, we chose a scoping review as the most fitting design since it proves valuable in surveying the evolving or emerging subjects in literature and pinpointing areas where there is limited information [42]. We followed the PRISMA guidelines for scoping reviews [43]. A systematic search of Medline, Embase, and the Cochrane Central Register of Controlled Trials using keywords and database-specific subject headings (last search date: 17 August 2023) was conducted. In addition, citation tracking was performed to review the reference lists and cited articles in the included studies. The full search strategy is listed in Supplemental Digital Content S1.

### 2.2. Eligibility Criteria

We included original articles reporting on two-armed studies with patients receiving postmastectomy radiotherapy compared to patients not receiving adjuvant irradiation following immediate autologous reconstruction. Non-original articles (i.e., editorials, letters, narrative reviews), case reports, conference abstracts, trial registry entries and one-armed studies were excluded. Studies that did not report objective numerical flap volume values were also excluded.

### 2.3. Outcomes

Free flap volume changes were measured through objective volumetric analysis (MRI, CT, superficial 3D imaging, mammometer) and reported as percentages or in cubic centimeters or milliliters. 

### 2.4. Study Identification and Data Extraction

**Figure 1:**
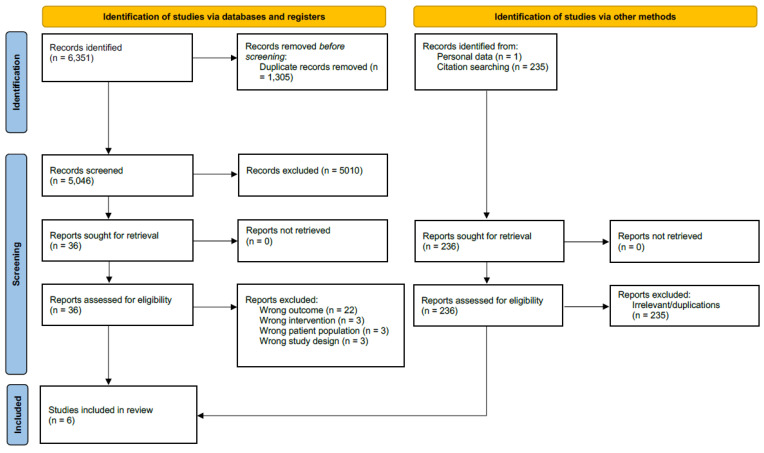
Preferred Reporting Items for Systematic review and Meta-analyses (PRISMA) 2020 flow diagram for new systematic reviews, including searches of databases, registers, and other sources. Cochrane CENTRAL, the Cochrane Central Register of Controlled Trial.

All retrieved references were exported to Endnote 20 (Endnote Version 20, Clarivate Analytics, 2020). Further study selection was performed in the web-based application Covidence (Veritas health Innovation, 2023; www.covidence.org, accessed on 31 August 2023). First, two reviewers independently screened all the references, and then, they screened the full text articles resulting from this search to determine eligibility. Disagreements on eligibility were solved via consensus or by a third reviewer. The Preferred Reporting Items for Systematic review and Meta-analyses (PRISMA) flow chart describing the screening process is shown in Figure 1 [44]. Data extraction for the selected studies was also performed independently by two reviewers using a predetermined spreadsheet and comparisons were made accordingly. Disagreements were again solved via consensus or by a third reviewer.

## 3. Results

### 3.1. Study Characteristics

The primary literature search returned 5046 articles. After following the aforementioned review process, a full-text screening of 35 articles led to the inclusion of a total of six studies. In the identified studies, a total of 462 women, with a mean age of 49 years, had undergone unilateral or bilateral mastectomies and immediate autologous reconstruction. All of the studies were two-armed studies directly comparing patients receiving adjuvant radiotherapy and patients not receiving radiotherapy. Four studies were retrospective analyses, and two studies were prospective cohort studies. Three used surface 3D imaging methods to assess flap volume, followed by two studies using CT and/or MRI measurement [35,36,37,38,39,40,45]. One study used a mammometer for the volume measurements [34]. Regarding radiation regimen, only two of the six studies reported the radiation dosage and fraction [34,38]. Table 1 presents the descriptive summary of the included studies. 

### 3.2. Types of Flaps

The most frequent flap type was a free deep inferior epigastric perforator flap (DIEP, 96%). A total of ten patients received a free profunda artery perforator flap (PAP, 3%), followed by four patients receiving free transversus rectus abdominis myocutaneous flaps (TRAM, 1%). One study with 42 patients used DIEP and TRAM flaps but did not report the exact number [38].

### 3.3. Impact of Adjuvant Radiotherapy on Flap Volume

Six two-armed studies reported on the effect of postmastectomy radiotherapy on flap volume changes. Of those, two studies found a significant reduction in flap volume following adjuvant irradiation, comparing a total of 173 patients. Four studies did not find a significant impact of radiotherapy, comparing a total of 289 patients.

Two of the six studies assessed the numerical flap volume, comparing irradiated and non-irradiated flaps; Chatterjee et al. reported that 12 months after reconstruction, the non-irradiated flaps maintained their initial volumes, as measured using mammometry, compared to a reduction of 7.89% of the initial volume in patients receiving adjuvant radiotherapy. The difference between the two groups was found to be non-significant (*p* ≥ 0.05) [34]. Myung et al. stated that at 12 to 18 months after reconstruction, the non-irradiated flaps showed a decrease in volume by 2.6%, as measured using computer tomography (CT), compared to a statistically significant volume reduction of 12.3% in the irradiated flaps (*p* ≤0.05) [38].

## 4. Discussion

Patients with autologous breast reconstruction after mastectomy experience a high satisfaction with their breasts and breast-related quality of life, which could potentially be influenced by adjuvant radiotherapy [46,47]. From an aesthetic standpoint, one of the primary surgical objectives of breast reconstruction is to achieve a balanced restoration of volume symmetrical to the contralateral side [48]. Since symmetrical breast volume represents a significant parameter for the outcome evaluation of breast reconstruction, a negative influence of adjuvant radiotherapy would be of clinical importance [40]. Assuming that postoperative radiotherapy considerably impacts free flap volume, delaying the reconstruction or opting for neoadjuvant radiotherapy would emerge as a valid alternative. In the scenario of preoperative radiotherapy, the complete avoidance of irradiating the reconstructed breast tissue could be achieved without compromising oncological safety [49]. Previous studies have shown that postmastectomy radiotherapy after breast reconstruction can lead to increased complication rates [26]. In this context, irradiation has been linked to a higher incidence of volume loss in the reconstructed breast [50,51,52]. A systematic review and meta-analysis by Liew et al. reported a significantly higher risk of developing a decrease in volume after adjuvant radiotherapy following autologous breast reconstruction [26]. The analysis primarily relied on subjective clinical assessments, combining fibrotic contracture and/or volume loss into one category. Notably, none of the studies included in the meta-analysis used objective volumetric outcome measurements. This identified the need for a more specific review. Our scoping review aims to summarize and compare existing literature concerning objective flap volume assessments in patients undergoing autologous breast reconstruction with postoperative radiotherapy.

### 4.1. The Importance of a Predictable Flap Volume

To preoperatively plan the required flap volume, understanding the extent to which this volume will ultimately be retained is crucial [40]. The objective of preoperative volume planning is to overcompensate the initial flap volume concerning its anticipated final volume. Although there have been some studies evaluating the residual postoperative flap volume, specific factors and their impacts have seldom been thoroughly examined [53]. Most volumetric assessment studies have focused on head and neck flap reconstruction. Particularly in cases of tongue reconstruction, flap volume remains an essential predictor of long-term functional outcomes, encompassing speech production and swallowing function [54,55]. These studies have reported a postoperative volume reduction in a wide range from 10 to 55% [56,57]. Sakamoto et al. examined the muscle:fat ratio and discovered that within flap tissue, muscle tissue undergoes a more significant reduction than fatty tissue [58]. The final fat volume at 12 months postoperatively was reported to be 85.5%, whereas the muscle tissue was reduced to almost 35% of its original value [58]. This outcome aligns with the literature since denervated muscle tissue in free flaps has been shown to undergo morphological changes that result in atrophy [59]. The observed variability in the reduction in fatty or muscle tissue suggests that the composition of the flap, and consequently the flap choice, will significantly impact postoperative volume loss. Additionally, factors such as postoperative body weight loss, recipient site location, and radiation fractionation schedule must be taken into account [56,60,61]. 

Unfortunately, even when considering all those factors, predicting the final volumetric outcome remains somewhat inaccurate and cannot be generalized across different patient populations. This is due to substantial differences in the primary flap used, recipient site location, radiation doses, and varying radiobiological characteristics. Furthermore, volumetric measurement methods differ for breast volume studies and head and neck reconstruction, further limiting the generalizability of existing literature.

### 4.2. Methods of Measuring Flap Volume

Various options exist for objectively measuring flap volume, each with distinct technical profiles. Magnetic resonance imaging is recognized to be the most accurate method for assessing flap volume, but its high cost may limit its application in research [62]. Nevertheless, due to its exceptional image quality, MRI scans are preferred, particularly when evaluating soft tissues like the breast [62]. It has been successfully used in the assessment of flap volume in head and neck reconstruction, alongside with CT scans [58,63,64,65,66]. In contrast to MRI, CT scans are generally more accessible, cost-effective, and proficient in accurately measuring tissue volume [67,68]. However, the main drawback is the exposure to ionizing radiation [62]. In addition to postoperative evaluation, CT scans facilitate preoperative flap planning and the mapping of perforators, effectively reducing morbidity and duration in perforator flap reconstruction [62,69]. A relatively new diagnostic tool for volume measurement is 3D surface imaging, which has gained increasing attention in breast volumetry research [70]. This technique offers the advantage of swiftly capturing breast geometry while being non-invasive, preserving the natural shape of the breast in an upright posture [71]. Moreover, surface measurements allow for a direct correlation with MRI results, establishing them as reliable and reproducible methods suitable for clinical applications [72]. It is important to note that 3D imaging tends to provide smaller breast volume measurements compared to MRI [70,72]. 

Surprisingly, one study in our review employed a mammometer for volumetric assessment [34,73]. To our knowledge, this is the sole study utilizing mammometry for flap volume assessment since its first description by Morris et al. [73]. Due to its lack of implementation as a standardized method and the absence of validation data, we consider this technique unreliable and do not recommend its use. Lastly, we did not find a study that used mammography images to assess flap volume. Mammography is the primary imaging modality used in regular breast cancer follow-ups [74]. As such, those images are widely accessible and mammography has been described as a useful option to assess breast volume [74,75]. While this could offer an interesting approach for future studies, the correlation between flap volume and mammography images needs validation.

### 4.3. Flap Volume after Breast Reconstruction and the Impact of Adjuvant Radiotherapy

Few studies have reported on volumetric outcomes following immediate autologous breast reconstruction in general. Park et al. observed an average volume reduction of around 10% two years postoperatively in patients receiving TRAM flap reconstruction without adjuvant radiotherapy [76]. When looking at the muscle:fat ratio, similar results to head and neck reconstruction were seen in TRAM flap breast reconstruction. Muscle tissue in TRAM flaps underwent a significant reduction of 70% after 15 months following the initial breast reconstruction in patients without adjuvant radiotherapy [77]. Studies evaluating the DIEP flap, often considered the workhorse flap, have reported volume losses ranging from a slight increase to a decrease of 26%, aligning with findings from adipose flaps used in head and neck reconstructive procedures [35,36,40]. Wilting et al. conducted a substantial study with 136 patients, assessing flap volumes over six months using superficial 3D imaging. They observed a flap volume reduction of 11.1%, primarily featuring DIEP flaps (91%) [40]. Kim et al. reported a more pronounced reduction of 19.9% one year after surgery and 26.2% two years postoperatively. Their analysis, primarily involving DIEP flaps (97%), utilized measurements derived from CT or MRI images [36]. Notably, these figures represent the average volume reduction in a mixed patient population with varying exposure to radiotherapy. In these studies, the primary loss of flap volume was observed within the initial year, followed by a continuous decline thereafter [35,36,40]. Extending the follow-up period could offer insights into potential further volume reduction and shed more light on the impact of adjuvant radiotherapy. Animal studies have indicated that irradiated tissues exhibit increased susceptibility to micro-thrombotic events, tissue necrosis, and decreased vascular density, primarily due to delayed flap revascularization, suggesting a probable negative impact on flap tissue. However, existing literature on the effect of radiotherapy remains contentious [78,79,80,81]. 

In our systematic literature search, we identified six two-armed studies directly comparing patients receiving adjuvant radiotherapy to those not receiving it. Interestingly, only two studies found a significant effect of radiotherapy on flap volume [36,38]. This finding was unexpected as it contradicts long-held beliefs. In the first study by Myung et al., flap volumes reduced by 12.3% after radiation compared to 2.6% in non-irradiated flaps 12–18 months postoperatively [38]. Of note, the baseline CT scans in this study were obtained at a minimum of three months postoperatively. While the impact of radiotherapy was statistically significant, caution is advised in interpreting the reported numerical values. The authors did not factor in the early volume loss in their study design, suggesting that the observed volume decrease within the initial three months might have been greater than documented. 

The second study, which also identified a significant negative impact, did not provide exact numerical values. Kim et al. utilized uni- and multivariable linear regression analysis to demonstrate statistical impact but did not present the absolute flap volume changes [36]. Among the remaining four studies, none found a significant difference. Among these, only one study provided precise numerical values measured using mammometry in 68 patients. At twelve months postoperatively, irradiated flaps exhibited an 8.9% volume reduction while non-irradiated flaps showed no volume reduction, although this difference did not reach statistical significance. Despite the specific investigation into the impact of radiotherapy in the selected studies, there was relatively limited reporting on radiation doses and fractionation schedules.

None of the included studies examined the potential impact of various radiation doses or alternative fractionation schedules as variables. Surprisingly, only two studies detailed their employed radiation strategies. Historically, adjuvant radiotherapy for the breast and chest wall followed a normofractionation approach with single doses ranging from 1.8 to 2.0 Gray (Gy) up to a total dose of 50 Gy [82]. This conventional fractionation schedule was based on the premise that healthy tissue is more susceptible to changes in fraction dose compared to cancer cells. Administering 50 Gy in 25 fractions aimed to spare the surrounding organs, thereby minimizing acute and long-term side effects while ensuring optimal tumor control. Recent data have revealed that breast cancer might respond differently to changes in dose per fraction compared to other cancers. Consequently, protracting the treatment over five weeks may not offer advantages. Studies have established moderately hypofractionated radiotherapy as the standard of care after breast-conserving surgery, replacing conventionally fractionated treatment over five weeks, with 40 Gy delivered in 15 fractions. This shift has not shown differences in tumor control but has presented similar or even better cosmetic outcomes such as reduced breast induration, skin toxicity, and breast edema [83]. Moreover, there are favorable data supporting the use of hypofractionation after mastectomy [84,85,86]. Wang et al. demonstrated equivalent oncological outcomes for patients undergoing postmastectomy radiotherapy (without reconstruction), with a significantly lower incidence of grade 3 skin toxicity after receiving 43.5 Gy in 15 fractions compared to 50 Gy in 25 fractions [84]. Additionally, Chang et al.’s study suggested that complications in implant-based breast reconstruction are notably influenced by high maximum doses, hinting at potential benefits of hypofractionated radiotherapy [87]. Meanwhile, Chung et al.’s study questioned the correlation between postmastectomy radiation dosage and complications following breast reconstruction.

It demonstrated a 27% complication rate among irradiated patients with autologous reconstruction, with a low incidence of major complications at 3%. Notably, the total radiation dose emerged as one of the independent risk factors for these major complications [88]. However, none of these referenced studies evaluated the impact of different radiation doses or fractionation regimens on changes in free flap volume. While there is substantial evidence suggesting potential benefits of hypofractionated radiotherapy in terms of complication rates, its influence on free flap volume needs further confirmation.

Assuming that postoperative radiotherapy significantly impacts free flap volume, neoadjuvant radiotherapy presents a potential alternative. The use of preoperative radiotherapy circumvents the irradiation of the reconstructed breast tissue.

### 4.4. Strengths and Limitations

Our systematic literature search enabled us to identify the current state of knowledge and provide an overview of the existing evidence on this topic. However, it is crucial to acknowledge that scoping reviews inherently have limitations as they aim to offer a general overview of a specific topic rather than conduct an in-depth analysis [89]. We acknowledge and summarize the limitations of this review. Notably, the studies we selected did not account for other potential variables that could introduce bias, such as postoperative weight loss and the specific regimens for adjuvant cancer treatment. These factors can significantly impact flap volume in the adjuvant context, and the analyses should have been adjusted accordingly [61,90]. Most importantly, only two studies reported radiation dose and fractionation. This could significantly impact the influence of radiotherapy on free flap volume and needs to be addressed separately in future studies. Specifically, the implementation of hypofractionation in the postmastectomy setting compared to (historically used) conventionally fractionated radiotherapy could yield some interesting aspects of this topic. The selected studies exhibit variability in patient numbers, follow-up timepoints, types of flaps, and measuring methods, contributing to high heterogeneity. Another issue in summarizing these studies was the use of non-uniform measurement tools for flap volume assessment, potentially invalidating direct comparisons between different techniques. Limited data reporting, especially regarding exact numerical values of respective flap volumes, rendered a meta-analysis unfeasible. Within this scoping review, only two studies demonstrated a statistically significant impact of radiotherapy on flap volume. However, data from animal studies, research in head and neck reconstruction, and clinical experiences strongly suggest a negative impact. Yet, confirming whether this translates to clinically significant differences in breast cancer requires robust prospective trials that measure flap volume via CT or MRI. None of the studies investigated the correlation between flap volume and patient satisfaction, highlighting the need for future studies to assess the actual importance of accurate flap volumes. Moreover, while the goal of achieving adequate breast volume ultimately targets breast symmetry, this may not solely determine patient satisfaction. Although patients may observe postoperative breast asymmetry, it does not necessarily result in dissatisfaction. The entire breast reconstruction process, inclusive of preoperative information, holds greater significance in influencing post-surgery patient sentiments and satisfaction with the overall outcome compared to the final aesthetic result of the reconstruction itself [27]. Therefore, solely focusing on objective breast symmetry might overlook crucial variables impacting patient-subjective outcomes.

## 5. Conclusions

Predictable flap and breast volumes remain crucial outcomes in immediate autologous breast reconstruction. We cannot confirm or contradict adjuvant radiotherapy’s impact on breast flap volume, and the limited body of evidence does not allow for a valid meta-analysis. One possible reason for the limited number of studies may be that a regular breast cancer follow-up typically does not necessitate volumetric evaluation. The primary imaging modality used, mammography, lacks validation for flap volume assessment [74]. Further studies are necessary to definitively assess the impact of adjuvant radiotherapy on free flap volume in immediate breast reconstruction and its correlation with patient satisfaction.

## Figures and Tables

**Table 1:** Descriptive summary of selected studies.

Author	Year	Study Type	Country	*n*	Age (Mean)	Significant Impact of RT on Flap Volume *	Radiation Dosage/Fractionation	Type of Flaps	Measurement Method	Follow-Up Time	Timing of Reconstruction
Chatterjee	2009	Prospective	United Kingdom	68	52	No	45 Gy/20 fractions	DIEP	Mammometer	N/A	Immediate
Craig	2018	Retrospective	USA	11	N/A	No	N/A	DIEP	3D superficial image	30 months	Immediate
Kim	2023	Retrospective	Korea	131	48	Yes	N/A	DIEP/TRAM	CT/MRI	26 months	Immediate
Lee	2023	Prospective	Korea	74	49	No	N/A	DIEP	3D superficial image	12 months	Immediate
Myung	2018	Retrospective	Korea	42	46.9	Yes	50 Gy/25 fractions	DIEP/TRAM	CT	12–18 months	Immediate
Wilting	2020	Retrospective	Netherlands	136	49.1	No	N/A	DIEP/SIEA/PAP	3D superficial image	12 months	Mixed **

DIEP, deep inferior epigastric perforator flap. PAP, profunda artery perforator flap. RT, radiotherapy. SIEP, superficial. inferior epigastric artery flap. TRAM, transversus rectus abdominis myocutaneous flap. * Statistical significance was defined at *p* ≤ 0.05. ** Mixed reconstruction timings including immediate, sequential, or delayed reconstruction.

## Data Availability

Data presented in this article are available in Table 1.

## Supplementary Materials

The following are available online at https://www.mdpi.com/article/10.3390/jcm13010217/s1, Supplemental Digital Content S1: Search strategy.
