# Supplementary material for: Impact of Adjuvant Radiotherapy on Free Flap Volume in Autologous Breast Reconstruction: A Scoping Review
Source: J Clin Med. 2023 Dec 29;13(1):217. doi: 10.3390/jcm13010217 (PMC10779607; doi:10.3390/jcm13010217)
Supplement: Supplementary file 1 [file jcm-13-00217-s001.zip › jcm-2737982-supplementary.pdf]

Supplemental Digital Content 1

Search strategy and study selection process

S. M. systematically searched Medline, Embase and the Cochrane Central Register of Controlled Trials (CENTRAL) using textwords and database-specific subject headings (last date of search: August XXX, 2023). The search strategy was initially drafted for PubMed, translated with the Polyglot Search Translator to the other databases and peer-reviewed by P.P. As a supplementary search, citation tracking was performed to review reference lists and citing articles of the included studies (see full strategy in Webappendix).

All retrieved references were exported to Covidence and duplicates were removed.

Two reviewers (SM, PP) independently screened all references resulting from the search. They then independently screened all potentially eligible articles in full-text for eligibility. Disagreements were solved by consensus/by a third reviewer.

Narrative

- Seed Papers: 10.1055/a-1947-8116 OR 10.1097/SAP.0000000000001462 OR 10.1097/00006534-200205000-00022 OR https://doi.org/10.1002/bjs.6693 OR 10.1097/PRS.0b013e3182729d33 OR 10.1016/j.bjps.2015.06.003 OR
  - Lee, Three-dimensional Volume Changes of the Reconstructed Breast Following DIEP Flap Breast Reconstruction, 10.1055/a-1947-8116
  - Craig, Three-dimensional Analysis of How Radiation Affects Deep Inferior Epigastric Perforator (DIEP) Flap Volume, Projection, and Position in Breast Cancer Reconstruction, 10.1097/SAP.0000000000001462
  - Rogers, Radiation Effects on Breast Reconstruction with the Deep Inferior Epigastric Perforator Flap, 10.1097/00006534-200205000-00022
  - Chatterjee, Effect of postoperative radiotherapy on autologous deep inferior epigastric perforator flap volume after immediate breast reconstruction, https://doi.org/10.1002/bjs.6693
  - Chang, Effects of Radiation Therapy for Breast Cancer Based on Type of Free Flap Reconstruction, 10.1097/PRS.0b013e3182729d33
  - Taghizadeh, Does post-mastectomy radiotherapy affect the outcome and prevalence of complications in immediate DIEP breast reconstruction? A prospective cohort study, 10.1016/j.bjps.2015.06.003
  - Motwani, The impact of immediate breast reconstruction on the technical delivery of postmastectomy radiotherapy, 2006
  - Williams, The effects of radiation treatment after TRAM flap breast reconstruction.
  - Radiation effects on breast reconstruction with the deep inferior epigastric perforator flap, 1997
  - Mirzabeigi, An assessment of the risks and benefits of immediate autologous breast reconstruction in patients undergoing postmastectomy radiation therapy, 2013
  - Taghizadeh, Does post-mastectomy radiotherapy affect the outcome and prevalence of complications in immediate DIEP breast reconstruction? A prospective cohort study, 2015

    - Terao, Postmastectomy radiation therapy and breast reconstruction with autologous tissue, 2017
    - Myung, Objective assessment of flap volume changes and aesthetic results after adjuvant radiation therapy in patients undergoing immediate autologous breast reconstruction, 2018
- The topic of this review was on patients suffering from breast neoplasms, who underwent free flap surgical reconstruction followed by adjuvant radiotherapy and particularly the effect of the latter on flap volume. Given this topic, a search strategy included the search blocks ("Breast Neoplasms") AND ("Surgical Flaps") AND ("Radiotherapy").
- The applied search strategy identified 12/12 seed papers. In order to further strengthen the search strategy, it was deemed worthwhile to conduct a thorough citation tracking as supplementary search technique.

Database searching

Pubmed Medline

Search date 15.08.2023; PubMed ALL 1956 to August 13, 2023; 2’733hits)

(Breast neoplasms[MeSH Terms]

OR

(Breast*[tw] OR Mamma*[tw] OR nipple*[tw] OR MAK[tw] OR “mammary gland*”[tw] OR mammilla*[tw] OR areola*[tw] OR “mammillary duct*”[tw] OR “lactiferous duct*”[tw] OR “lactiferous sinus*”[tw] OR “milk duct*”[tw]

AND

carcino*[tw] OR adenocarcino*[tw] OR cancer*[tw] OR neoplas*[tw] OR tumor*[tw] OR tumour*[tw] OR malignan*[tw] OR dysplas*[tw] OR adenom*[tw] OR “paget diseas*”[tw] OR mass*[tw] OR metastas*[tw] OR lesion*[tw] OR “BRCA1 mutat*”[tw] OR “BRCA2 mutat*”[tw] OR DCIS[tw] OR IDC[tw] OR ILC[tw] OR IBC[tw]))

AND

(Surgical Flaps[MeSH Terms] OR Plastic Surgery Procedures[MeSH Terms] OR Surgery/Plastic[MeSH Terms]

OR

(surgical[tw] OR "free"[tw] OR "tissue"[tw] OR "deep inferior epigastric"[tw] OR "transverse rectus abdominis myocutaneous"[tw] OR "superficial inferior epigastric artery"[tw] OR "superior gluteal artery perforator"[tw] OR "inferior gluteal artery perforator"[tw] OR "transverse upper gracilis"[tw] OR "profunda artery perforator"[tw] OR "lateral thigh"[tw] OR DIEP[tw] OR TRAM[tw] OR ms-TRAM[tw] OR SIEA[tw] OR SGAP[tw] OR IGAP[tw] OR TUG[tw] OR PAP[tw] OR TRAM[tw] OR TRAM[tw] OR "medial thigh"[tw] OR fasciocutaneous[tw] OR fascio-cutaneous[tw] OR muscle[tw] OR myocutaneous[tw] OR myo-cutaneous[tw] OR musculocutaneous[tw] OR musculo-cutaneous[tw] OR muscular-cutaneous[tw] OR perforator[tw]

AND

flap[tw] OR flaps[tw] OR graft[tw] OR grafts[tw] OR "flap repair*"[tw] OR "flap reconstruction*"[tw] OR "graft repair*"[tw] OR "graft reconstruction*"[tw]))

AND

(Radiotherapy[MeSH Terms]

OR

radiotherap*[tw] OR radiation*[tw] OR irradiat*[tw] OR nonirradiat*[tw] OR non-irradiat*[tw] OR preirradiat*[tw] OR pre-irradiat*[tw] OR postirradiat*[tw] OR post-irradiat*[tw])

Search Term PubMed

("breast neoplasms"[MeSH Terms] OR (("breast*"[Text Word] OR "mamma*"[Text Word] OR "nipple*"[Text Word] OR "MAK"[Text Word] OR "mammary gland*"[Text Word] OR "mammilla*"[Text Word] OR "areola*"[Text Word] OR "mammillary duct*"[Text Word] OR "lactiferous duct*"[Text Word] OR "lactiferous sinus*"[Text Word] OR "milk duct*"[Text Word]) AND ("carcino*"[Text Word] OR "adenocarcino*"[Text Word] OR "cancer*"[Text Word] OR "neoplas*"[Text Word] OR "tumor*"[Text Word] OR "tumour*"[Text Word] OR "malignan*"[Text Word] OR "dysplas*"[Text Word] OR "adenom*"[Text Word] OR "paget diseas*"[Text Word] OR "mass*"[Text Word] OR "metastas*"[Text Word] OR "lesion*"[Text Word] OR "brca1 mutat*"[Text Word] OR "brca2 mutat*"[Text Word] OR "DCIS"[Text Word] OR "IDC"[Text Word] OR "ILC"[Text Word] OR "IBC"[Text Word]))) AND ("surgical flaps"[MeSH Terms] OR "plastic surgery procedures"[MeSH Terms] OR "plastic surgery procedures"[MeSH Terms] OR (("surgical"[Text Word] OR "free"[Text Word] OR "tissue"[Text Word] OR "deep inferior epigastric"[Text Word] OR "transverse rectus abdominis myocutaneous"[Text Word] OR "superficial inferior epigastric artery"[Text Word] OR "superior gluteal artery perforator"[Text Word] OR "inferior gluteal artery perforator"[Text Word] OR "transverse upper gracilis"[Text Word] OR "profunda artery perforator"[Text Word] OR "lateral thigh"[Text Word] OR "DIEP"[Text Word] OR "TRAM"[Text Word] OR "ms-TRAM"[Text Word] OR "SIEA"[Text Word] OR "SGAP"[Text Word] OR "IGAP"[Text Word] OR "TUG"[Text Word] OR "PAP"[Text Word] OR "TRAM"[Text Word] OR "TRAM"[Text Word] OR "medial thigh"[Text Word] OR "fasciocutaneous"[Text Word] OR "fascio-cutaneous"[Text Word] OR "muscle"[Text Word] OR "myocutaneous"[Text Word] OR "myo-cutaneous"[Text Word] OR "musculocutaneous"[Text Word] OR "musculo-cutaneous"[Text Word] OR "muscular-cutaneous"[Text Word] OR "perforator"[Text Word]) AND ("flap"[Text Word] OR

"flaps"[Text Word] OR "graft"[Text Word] OR "grafts"[Text Word] OR "flap repair*"[Text Word] OR "flap reconstruction*"[Text Word] OR "graft repair*"[Text Word] OR "graft reconstruction*"[Text Word]))) AND ("radiotherapy"[MeSH Terms] OR ("radiotherap*"[Text Word] OR "radiation*"[Text Word] OR "irradiat*"[Text Word] OR "nonirradiat*"[Text Word] OR "non irradiat*"[Text Word] OR "preirradiat*"[Text Word] OR "pre irradiat*"[Text Word] OR "postirradiat*"[Text Word] OR "post irradiat*"[Text Word]))
